# Local Sharing as a Predominant Determinant of Synaptic Matrix Molecular Dynamics

**DOI:** 10.1371/journal.pbio.0040271

**Published:** 2006-08-15

**Authors:** Shlomo Tsuriel, Ran Geva, Pedro Zamorano, Thomas Dresbach, Tobias Boeckers, Eckart D Gundelfinger, Craig C Garner, Noam E Ziv

**Affiliations:** 1 The Rappaport Family Institute for Research in the Medical Sciences, Technion Faculty of Medicine, Haifa, Israel; 2 The Department of Physiology, Technion Faculty of Medicine, Haifa, Israel; 3 Department of Psychiatry and Behavioral Science, Nancy Pritzker Laboratory, Stanford University, Palo Alto, California, United States of America; 4 Institute of Anatomy and Cell Biology II, University of Heidelberg, Heidelberg, Germany; 5 Institute of Anatomy and Cell Biology, University of Ulm, Ulm, Germany; 6 Department of Neurochemistry and Molecular Biology, Leibniz Institute for Neurobiology, Magdeburg, Germany; Howard Hughes Medical Institute, United States of America

## Abstract

Recent studies suggest that central nervous system synapses can persist for weeks, months, perhaps lifetimes, yet little is known as to how synapses maintain their structural and functional characteristics for so long. As a step toward a better understanding of synaptic maintenance we examined the loss, redistribution, reincorporation, and replenishment dynamics of Synapsin I and ProSAP2/Shank3, prominent presynaptic and postsynaptic matrix molecules, respectively. Fluorescence recovery after photobleaching and photoactivation experiments revealed that both molecules are continuously lost from, redistributed among, and reincorporated into synaptic structures at time-scales of minutes to hours. Exchange rates were not affected by inhibiting protein synthesis or proteasome-mediated protein degradation, were accelerated by stimulation, and greatly exceeded rates of replenishment from somatic sources. These findings indicate that the dynamics of key synaptic matrix molecules may be dominated by local protein exchange and redistribution, whereas protein synthesis and degradation serve to maintain and regulate the sizes of local, shared pools of these proteins.

## Abstract 2

To understand processes involved in synaptic maintenance, the authors examine the loss, redistribution, reincorporation and replenishment dynamics of two key synaptic proteins, Synapsin I and ProSAP2/Shank3.

## Introduction

Chemical synapses are specialized sites of cell-cell contact designed for the transmission of signals between neurons and their respective targets. Until recently, not much was known on the life span of individual synaptic connections, in particular of those found within the mammalian central nervous system (CNS). Recent in vivo imaging studies, however, indicate that many, perhaps the majority of CNS synaptic connections are remarkably persistent, exhibiting life spans of weeks, months, and perhaps years [1–3].

What mechanisms allow these important devices to persist for such long durations? This is a crucial question on several levels: at a very basic level, appropriate CNS function clearly depends on the presence of functional synapses. A different level, however, relates to the persistence of activity-dependent changes to the function of an individual synapse (collectively referred to as synaptic plasticity): for these changes to persist, it is not sufficient that the synapse simply persists; the synapse also has to somehow preserve the functional characteristics that reflect its physiological history.

Electron microscopic (EM) analysis of CNS synapses has revealed that the plasma membrane of the presynaptic compartment contains an electron-dense thickening that is juxtaposed and aligned with an electron-dense thickening of the postsynaptic membrane. The latter, known as the postsynaptic density (PSD), contains specialized molecules that form an elaborate molecular cytoskeletal matrix (cytomatrix) in which glutamate receptors are embedded [4]. Similarly, the presynaptic specialization, known as the active zone (AZ) contains a dense meshwork of structural proteins that is known as the cytoskeletal matrix associated with the active zonal membrane (CAZ). Facing the cytoplasmic aspect of the CAZ are numerous synaptic vesicles that are enmeshed in a fine matrix of proteins comprised primarily of microfilaments and the synaptic vesicle-associated protein Synapsin, which are thought to hold them together and keep them at the presynaptic region [5,6].

Presynaptic and postsynaptic specializations are not closed compartments but are continuous, to various degrees, with the axonal or dendritic cytoplasm and membrane. Despite this continuity, presynaptic boutons and postsynaptic compartments of shaft and spine synapses manage to maintain their unique structural organization. If synapses were static structural specializations, this would not be very remarkable. However, recent studies indicate that some components of synaptic matrices exhibit considerable dynamics, which are often accelerated by synaptic activation. At the presynaptic side, activity was shown to induce the rapid redistribution of proteins such as actin [7,8], Synapsin [9,10], Clathrin [11], and Rab3 [12]. On the postsynaptic side, it has been shown that neurotransmitter receptors continuously move between intrasynaptic and extrasynaptic pools and that these rapid dynamics are strongly affected by synaptic activity [13]. Furthermore, a number of studies have reported that certain PSD scaffolding molecules are continuously exchanged with molecules from extrasynaptic sources [14–21] and that activity can, in some cases, significantly enhance these molecular dynamics [14,18,20].

Given the dynamics exhibited by many synaptic molecules, it may be reasonable to surmise that the molecular structure of synaptic specializations at any point in time is the net outcome of processes that promote the assimilation of synaptic molecules into well-organized multimolecular complexes and forces that promote the loss of these molecules and consequently, lead to a reduction in complex size, organization, and stability [22]. Thus, an important step toward a better understanding of synaptic structure and maintenance is to obtain a better grasp of the processes and forces involved in the continuous assembly and disassembly of multimolecular complexes at synaptic junctions. Specifically, it is essential to address the following questions: What are the rates at which key synaptic proteins are lost from and reincorporated into individual synaptic structures? What happens to molecules lost from synapses? Are they necessarily degraded? Alternatively, are they reused? If so, are they reused by the same synapses or redistributed among and incorporated into nearby synapses? Are these processes use-dependent, i.e., are they accelerated by synaptic activity? To what degree is protein loss compensated for by local protein synthesis and by replenishment from somatic sources? How do these replenishment rates compare with local dynamics?

As a first step toward a better understanding of processes involved in the continuous assembly and disassembly of multimolecular complexes at individual CNS synapses, we have studied the loss, redistribution, reincorporation, and replenishment dynamics of Synapsin I and ProSAP2/Shank3, two prominent components of the presynaptic matrix and PSD, respectively. To that end we expressed EGFP and photoactivatable GFP-tagged variants of these molecules in cultured rat hippocampal neurons, and used fluorescence recovery after photobleaching (FRAP), photoactivation, and time-lapse confocal microscopy to explore the rates at which these molecules are lost from and reincorporated into individual synaptic specializations and to study the fate of molecules lost from such structures, the rates at which these synapses are replenished with molecules from somatic sources, and the dependence of these processes on protein synthesis and degradation. Our findings indicate that the dynamics of molecules within multimolecular complexes at individual CNS synapses may be dominated primarily by the continuous exchange and redistribution of synaptic proteins among nearby synapses whereas protein synthesis and degradation may constitute slower, second-order processes that serve to maintain and regulate the size of local, shared pools of synaptic matrix proteins.

## Results

### GFP:Synapsin I Loss and Reincorporation Rates at Individual Presynaptic Boutons

**Figure 1:**
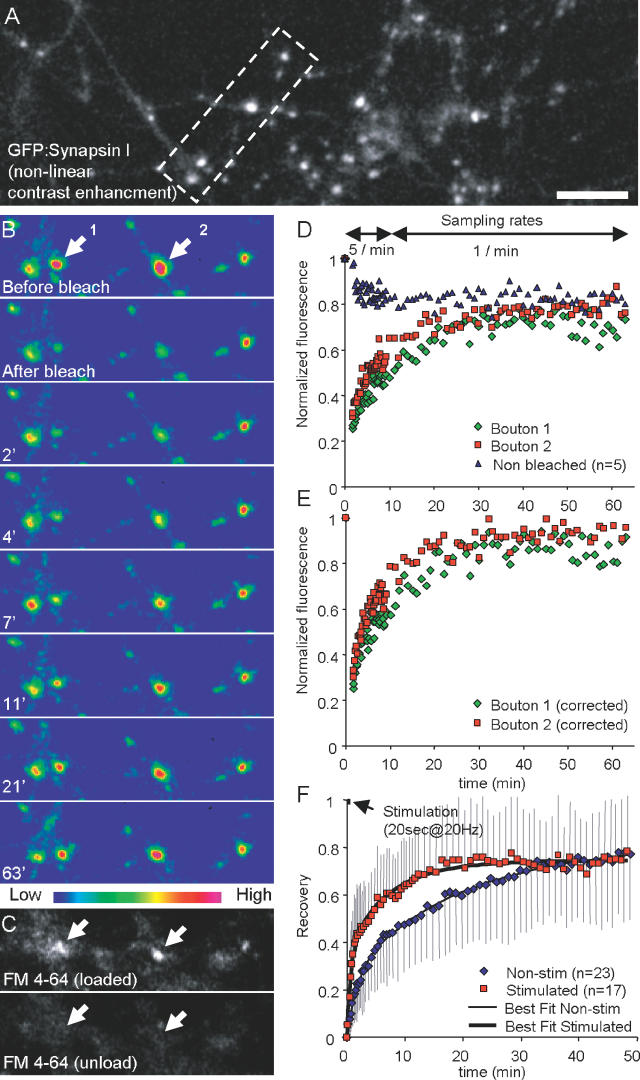
Loss and Reincorporation Rates of GFP:Synapsin I at Individual Synaptic Boutons (A) Axons of neurons expressing GFP:Synapsin I. Presynaptic boutons appear as bright puncta along faintly fluorescent axons. The contrast was enhanced nonlinearly in this figure to emphasize axonal fluorescence and demonstrate that the boutons photobleached here did not reside along the same axonal segment. Bar, 10 μm. (B) Two boutons (arrows) were selectively photobleached by high-intensity laser light, reducing their fluorescence to approximately 30% of their nominal values. Fluorescence recovery at these sites was then followed by time-lapse imaging, initially at 20-s intervals and later at 1-min intervals. GFP:Synapsin I fluorescence levels shown in false color according to color scale near bottom. (C) At the end of the experiment, presynaptic boutons were labeled (loaded) with FM4–64 by field stimulation (30sec@10hz) followed by unloading (120sec@10hz) to verify the functionality of the photobleached boutons (bottom panels). Note that both photobleached boutons exhibited a capacity for evoked endocytosis and exocytosis of FM4–64. Only boutons that exhibited such a capacity were included in our analysis. Same region as that enclosed in rectangle in (A). Times given in minutes. (D) Fluorescence recovery time course for photobleached boutons in (A) as well as the mean fluorescence of five nonphotobleached boutons in the same field. Note the gradual reduction of fluorescence in these boutons and its dependence on the sampling rate, indicating that illumination applied during ongoing imaging induces some photobleaching that should be corrected for. (E) Fluorescence recovery time course after correcting for ongoing photobleaching. (F) Loss and reincorporation of GFP:Synapsin I molecules at synapses are accelerated by synaptic activity. Neurons were stimulated for 20 s at 20 Hz immediately after collecting the first postbleach images. Despite the brief duration of this stimulation episode, fluorescence recovery was accelerated significantly in comparison to recovery in matched, nonstimulated preparations. Data shown are mean ± standard deviation for all photobleached boutons after normalization as described in Materials and Methods. One-sided error bars only are shown in sake of clarity. The data were fit to a model assuming two pools with different recovery kinetics as described in the text. All experiments were performed in the presence of CNQX (10 μM) and AP-5 (50 μM) to minimize spontaneous activity.

Synapsin I is a member of the Synapsin family of neuron-specific proteins that are thought to retain the reserve pool of synaptic vesicles within presynaptic boutons by binding to phospholipid and protein constituents of the synaptic vesicles on the one hand and to cytoskeletal components, such as actin and fodrin, on the other [23,24]. To study the rates at which Synapsin I is lost from and reincorporated into presynaptic structures, we expressed Synapsin I tagged with green fluorescent protein [9,10] (GFP:Synapsin I) in hippocampal neurons obtained from newborn rats and grown in culture for at least 2 wk. Rates of Synapsin I loss and reincorporation were determined by FRAP of GFP:Synapsin I at individual presynaptic boutons [25]. To that end, neurons expressing GFP:Synapsin I were mounted on the stage of a custom-built confocal microscope system and perfused slowly with a physiological saline solution. The chamber and objective of the inverted microscope were heated to 37 to 38 °C, resulting in intrachamber temperatures of 33 to 35 °C. As synaptic activity leads to reversible Synapsin dispersal [9,10], the glutamate receptor blockers 6-cyano-7-nitroquinoxaline-2,3-dione (CNQX, 10 μM) and 2-amino-5-phosphonopentanoic acid (AP-5, 50 μM) were added during these experiments to the perfusion solution to block spontaneous network activity. After collecting baseline images, three to five fluorescent puncta representing single boutons were selectively bleached by high-intensity 488-nm laser light, using an acousto-optical tunable filter (AOTF) to selectively illuminate regions of 1.5 × 1.5 μm centered on these boutons. Care was taken to photobleach boutons residing on separate axonal segments as shown in Figure 1. The boutons were photobleached under control of a computer program until their fluorescence was reduced to approximately 30% of their initial level, after which fluorescence recovery (signifying the loss of bleached molecules from the presynaptic bouton and the incorporation of unbleached molecules from outside the photobleached region) was monitored by automated time-lapse confocal microscopy (Figure 1B), initially at high sampling rates (5/min) and subsequently at slower rates (1/min). Focus drift was minimized during these recordings by performing an automated focusing procedure before each image stack was acquired.

As shown in Figure 1D, fluorescence recovery was usually gradual, and seemed to plateau after about 40 min. In most cases, however, the fluorescence did not fully recover to its initial value even after several hours. Although incomplete recovery is often interpreted to suggest the existence of a stable (immobile) pool of the tagged protein [25], incomplete recovery could also be due to other factors, such as ongoing photobleaching during the time-lapse recording of fluorescence recovery. Indeed, fluorescence of nonbleached GFP:Synapsin puncta exhibited reductions that depended on the sampling rates during the time-lapse session (Figure 1D) indicating that final fluorescence levels represented a balance between ongoing photobleaching and recovery processes. We thus corrected the data for the photobleached boutons by dividing their normalized fluorescence by the normalized fluorescence of nonbleached boutons at each time point. When this correction was applied, fluorescence recovery appeared to be much more complete (Figure 1E).

The time resolution in these experiments did not allow us to measure the extent of fluorescence recovery during the first few seconds after the photobleaching procedure. To determine if significant recovery occurs over this time-scale, we performed line-scan imaging of individual boutons during which the illumination intensity was transiently increased by a factor of 50. These experiments revealed little recovery over this time-scale except for a minor component that recovered within less than 1 s of the bleaching procedure, probably representing a pool of cytosolic GFP:Synapsin within the bleached volume (Figure S1A).

To verify that the bleaching procedure did not impair the functionality of the photobleached presynaptic boutons, field stimulation (30sec@10Hz) was used to label all boutons in the fields of view with the fluorescent functional endocytosis marker *N*-(3-triethylammoniumpropyl)-4-(*p*-dibutylaminostyryl)pyridinium, dibromide (FM4–64, 15 μM). Although stimulation lead to a temporary dispersal of GFP:Synapsin I puncta [9,10], GFP:Synapsin I clusters reformed within minutes, enabling us to assess the degree of colocalization with FM4–64 puncta (Figure 1C). In agreement with previous studies [26–28], presynaptic boutons typically remained functional after the photobleaching procedure.

In most experiments, fluorescence recovery was monotonous as in Figure 1D and 1E. Occasionally, however, we noted abrupt steps in the recovery process, that were often associated with the merging of fluorescent mobile puncta with the photobleached bouton, or the splitting of the photobleached bouton into two or more separate puncta. These phenomena did not seem to be a direct effect of the photobleaching procedure, because similar phenomena were observed at unbleached boutons as well. Although the incorporation of mobile packets of presynaptic constituents has been documented in several studies [28,29] recovery of GFP:synapsin fluorescence occurred in most cases without an obvious involvement of discernable mobile packets.

### Activity Dependence of GFP:Synapsin I Loss and Reincorporation Rates

To determine how GFP:Synapsin I loss and reincorporation rates are affected by synaptic activity we performed FRAP experiments similar to those described above, except that here the recovery phase was preceded by synaptic activation. As activity leads to rapid Synapsin dispersion, continuous stimulation would have greatly complicated the interpretation of FRAP experiments. We thus established the following experimental procedure: Selected boutons were photobleached as described above. After obtaining the first postphotobleaching image, the preparations were stimulated once for 20 s at 20Hz, and fluorescence recovery kinetics were recorded by time-lapse imaging. Here too, glutamate receptor blockers were added to the perfusion solution to eliminate spontaneous activity.

Data from all experiments was pooled and compared, including only presynaptic GFP-Synapsin puncta that exhibited a capacity to recycle FM4–64 at the end of the experiment and that were not observed to split or incorporate mobile fluorescent material packets during the recovery phase. Data were not corrected for ongoing bleaching because the stimulation procedure affected the fluorescence of unbleached boutons and thus precluded the applicability of the correction procedure described above.

As shown in Figure 1F, fluorescence recovery in stimulated preparations occurred much faster than fluorescence recovery in nonstimulated preparations, despite the temporary fluorescence reduction caused by the brief stimulation episode (63% recovery: nonstimulated 23 min, stimulated 10 min; *n* = 23 for nonstimulated boutons, *n* = 17 for stimulated boutons, 13 preparations). However, the extent and kinetics of fluorescence recovery were quite variable within each group, as evident from the large error bars of Figure 1F. To determine if the apparent differences between the groups were statistically significant, we fit the recovery curve of each photobleached bouton to a recovery function that assumed two mobile pools of GFP:Synapsin I with different recovery kinetics. A computer program was then used to find the best fit to two time constants and to the relative sizes of the two pools (3 degrees of freedom). Differences between time constants in the two groups did not reach statistical significance (fast pool: 2.3 ± 1.4 versus 1.5 ± 1.3 min; slow pool 87 ± 112 versus 88 ± 114 min, nonstimulated and stimulated, respectively). However, the fractional size of the relatively mobile pool was significantly larger in the stimulated group (0.53 ± 0.21 stimulated versus 0.31 ± 0.18 nonstimulated, *p* < 0.005, Student's one-tailed *t*-test). These findings indicate that the faster recovery kinetics observed after a brief stimulation train result from an increase in the size of a relatively mobile pool of Synapsin I at the expense of a less mobile pool.

### Redistribution of Synapsin I among Nearby Boutons

What happens to Synapsin molecules after they dissociate from synaptic sites? It is often assumed that these molecules are subsequently degraded. However, an alternative possibility is that molecules that dissociate from one presynaptic locus are reincorporated into presynaptic structures at nearby loci, effectively resulting in the continuous redistribution of Synapsin among neighboring synapses. To determine if this occurs, we took advantage of the recently developed photoactivatable variant of GFP [30] (PA-GFP) to instantaneously “tag” the pool of Synapsin at one synaptic site and follow the fate of these molecules over time. To that end, we substituted the GFP moiety of GFP:Synapsin I with PA-GFP and transfected neurons with this fusion protein. As PA-GFP is practically invisible before activation, some method was necessary to visualize the transfected neurons and their synaptic structures before photoactivation. We thus co-expressed cyan fluorescent protein (CFP) with the PA-GFP–tagged Synapsin. CFP fluorescence was used to locate putative presynaptic sites, identified as varicosities along axons. Then, PA-GFP was activated in a single varicosity by high-intensity illumination at 405 nm confined to a very small region (about 3 × 3μm) centered on the selected varicosity. Time-lapse imaging was then used to follow the redistribution of the photoactivated PA-GFP:Synapsin molecules. In order to avoid the confounding effects of activity, all experiments were performed in the presence of glutamate receptor antagonists as described above.

**Figure 2:**
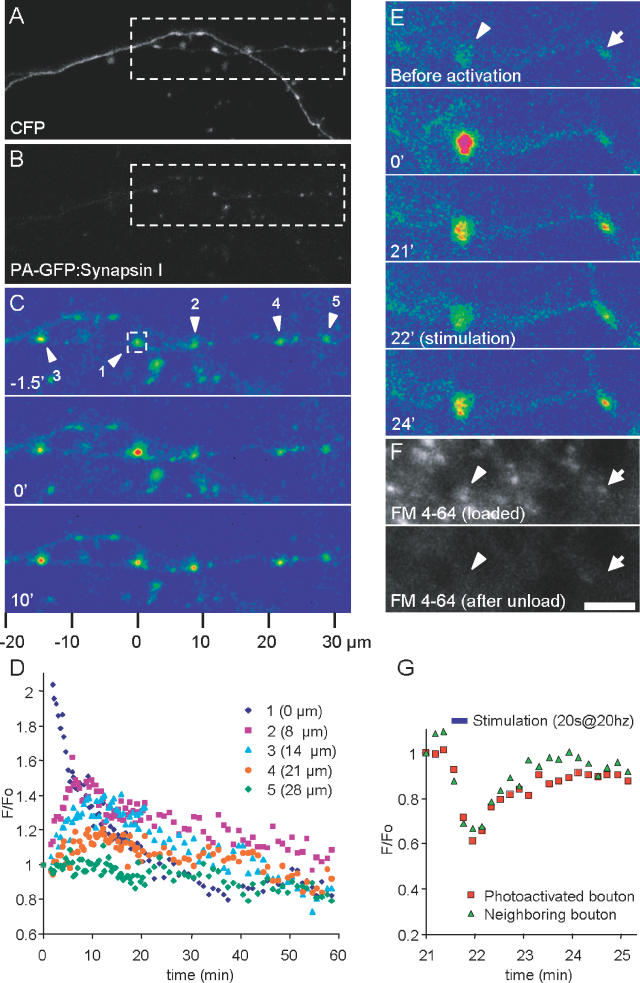
Synapsin I Lost from One Bouton Is Incorporated into Adjacent Boutons An axonal segment expressing both CFP (A) and PA-GFP:Synapsin I (B). (C) Higher magnification of region enclosed in rectangle in (B). PA-GFP:Synapsin I within a single bouton was photoactivated by selective illumination of this bouton at 405 nm (*t* = 0). Within 10 min of photoactivation, fluorescence at this bouton had decreased, whereas fluorescence at adjacent boutons (distances of 8 to 21 μm) had increased significantly. PA-GFP:Synapsin I fluorescence encoded in pseudo-color as in Figure 1. (D) Quantification of fluorescence changes following photoactivation at photoactivated and adjacent boutons. Note the rapid reduction of fluorescence at the photoactivated site and the concomitant (but transient) increases recorded at adjacent boutons that were most prominent at boutons nearest to the photoactivation site. (E) Synapsin lost from one bouton is incorporated into the presynaptic matrix of adjacent boutons. Two presynaptic boutons along an axonal segment expressing PA-GFP:Synapsin I. At time *t* = 0, PA-GFP:Synapsin I within one bouton (arrowhead) was photoactivated by selective illumination at 405 nm, and the redistribution of the photoactivated material was followed by time-lapse microscopy. At 21 min after photoactivation, a brief (20s@20Hz) stimulation train was delivered, leading to the transient dispersion of PA-GFP:Synapsin I at the photoactivated bouton as well as at the second bouton that had incorporated some of photoactivated PA-GFP:Synapsin I (arrow). PA-GFP:Synapsin I fluorescence encoded in pseudocolor as in Figure 1. (F) FM4–64 labeling at the end of the experiment confirmed that both sites were functional presynaptic boutons. Bar, 5 μm. (G) Normalized fluorescence of both boutons before, during, and after the stimulation episode. Note that PA-GFP:Synapsin I in the neighboring bouton exhibited activity induced dispersion just like Synapsin in the photoactivated bouton, indicating that the PA-GFP:Synapsin that had migrated to it had become incorporated into its presynaptic matrix.

One such experiment is shown in Figure 2A–2D. In this experiment, PA-GFP:Synapsin I within a single varicosity was activated moderately (F/F_0_ ≈ 2), and time-lapse fluorescence microscopy was then used to record the redistribution of the activated molecules. The fluorescence at the activated site decayed with kinetics similar to those observed for GFP:Synapsin I in the FRAP experiments described above (Figure 1). As fluorescence at the activated site decayed, transient increases in fluorescence were recorded in nearby varicosities, suggesting that material lost from the activated site was transiently incorporated into the presynaptic matrix of nearby synapses (Figure 2B). This effect was detectable at synapses residing at distances of up to 20 to 40 μm from the activated boutons, depending on the degree of activation and the relative sizes of the activated varicosities (11 separate experiments).

Given that varicosities represent sites of enlarged axonal diameter, any soluble fluorescent molecule would generate larger fluorescence signals at such sites simply due to geometrical considerations (as evident in CFP fluorescence images of such sites; Figure 2A). This raises the possibility that the increased fluorescence of activated PA-GFP:Synapsin observed at neighboring sites represents soluble PA-GFP:Synapsin rather than PA-GFP:Synapsin that had become incorporated into the presynaptic matrix. To determine if photoactivated PA-GFP:Synapsin had incorporated into the presynaptic matrix of neighboring boutons, we took advantage of the fact that stimulation leads to the temporary dispersion of presynaptic matrix-associated Synapsin [9,10]. To that end, we stimulated preparations for 20 s at 20 Hz approximately 20 min after photoactivating a single varicosity and examined if this led to dispersion of the activated PA-GFP:Synapsin that accumulated at neighboring presynaptic sites (three separate experiments). As shown in Figure 2E–2G, stimulation led to the reversible dispersion of punctate fluorescence at both photoactivated and neighboring sites, indicating that at least part of the PA:GFP-Synapsin that had migrated to neighboring boutons had become incorporated into their presynaptic matrix, strongly suggesting that Synapsin molecules are continuously interchanged among nearby presynaptic boutons at time-scales of tens of minutes.

### ProSAP2 Loss and Reincorporation Rates at Individual Postsynaptic Sites

ProSAP2 is a member of the ProSAP/Shank protein family, which are major constituents of glutamatergic synapse PSDs. These multidomain proteins interact directly and indirectly with a large number of postsynaptic proteins as well as with the actin-based cytoskeleton and have been suggested to serve as “master organizers” of postsynaptic cytoarchitecture [31–33]. Furthermore, ProSAP/Shank degradation was previously shown to be strongly affected by synaptic activity levels [34,35] and thus it seemed that these molecules may be particularly well suited to study the molecular dynamics of PSD maintenance.

**Figure 3:**
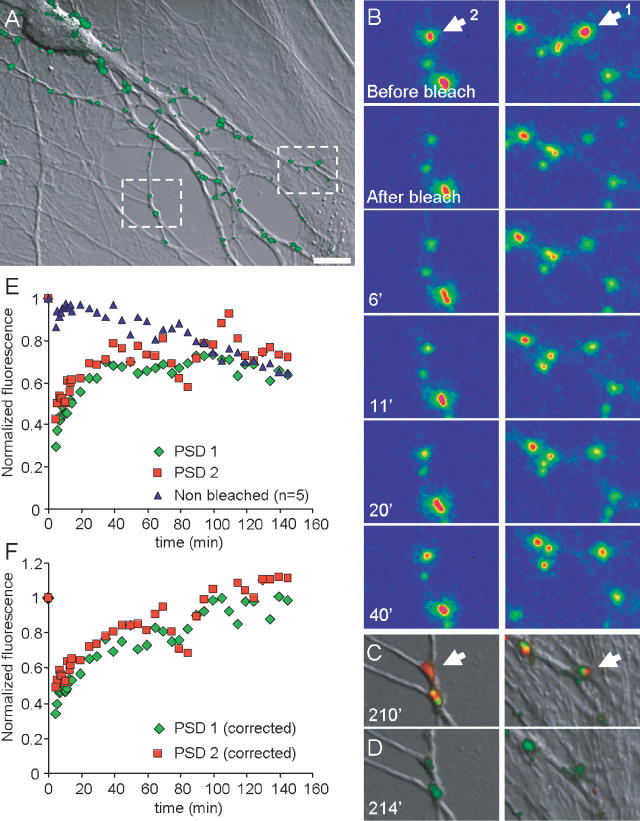
Loss and Reincorporation Rates of GFP:ProSAP2 at Individual Postsynaptic Sites (A) A fluorescence image of a neuron expressing GFP:ProSAP2 overlaid onto a DIC image of the same region. Postsynaptic sites appear as green puncta. Bar, 10 μm. (B) Two postsynaptic sites (arrows) were selectively photobleached by high-intensity laser light, reducing their fluorescence to approximately 30% to 40% of their nominal values. Fluorescence recovery at these sites was then followed by time-lapse imaging. Same regions as those enclosed in rectangles in (A). GFP:ProSAP2 fluorescence levels shown in false color as in Figure 1. (C and D) At the end of the experiment, presynaptic boutons in the field were labeled with FM4–64 (as described for Figure 1) to verify the synaptic identity of the photobleached GFP:ProSAP2 puncta. Note that the photobleached puncta (green) were juxtaposed to presynaptic boutons (red) that exhibited a capacity for evoked endocytosis (C) and exocytosis (D) of FM4–64. Only puncta with functional presynaptic counterparts were included in our analysis. (E) Fluorescence recovery time course for the photobleached GFP:ProSAP2 clusters shown in (B) as well as the mean fluorescence of five nonphotobleached clusters in the same field. (F) Fluorescence recovery time course after correcting for ongoing photobleaching.

We have previously found [17] that ProSAPs/Shanks exhibit high loss and incorporation rates at individual PSDs. However, these experiments were carried out in relatively immature neurons (9 to 10 d in vitro), and fluorescence recovery was followed for relatively short durations (minutes). We thus found it necessary to perform a detailed analysis in more mature neurons, after synaptogenesis-associated dynamics have subsided. To that end, we expressed a GFP-tagged variant of ProSAP2 (GFP:ProSAP2) in cultured hippocampal neurons (transfection on day 10 in vitro), and waited until days 16 to 24 in vitro to perform FRAP experiments. As reported previously [17], GFP:ProSAP2 was targeted correctly to postsynaptic sites and exhibited a punctate expression pattern along dendrites (Figure 3). Computer-controlled photobleaching of selected puncta, presumably representing individual PSDs followed by time-lapse recording of fluorescence recovery revealed that the recovery process was nearly complete within about 2 to 4 h (Figure 3B, 3E, and 3F). The synaptic identity of all photobleached puncta was examined by labeling functional presynaptic boutons with FM4–64 at the end of the experiments as shown in Figure 3C and 3D, and only photobleached puncta juxtaposed against functional presynaptic boutons were included in our analysis. Line-scan FRAP imaging, performed as described above, revealed little recovery over a time-scale of several seconds except for a very minor component that recovered within less than 1 s of the bleaching procedure, probably representing a pool of cytosolic GFP:ProSAP2 within the bleached volume (Figure S1C).

**Figure 4:**
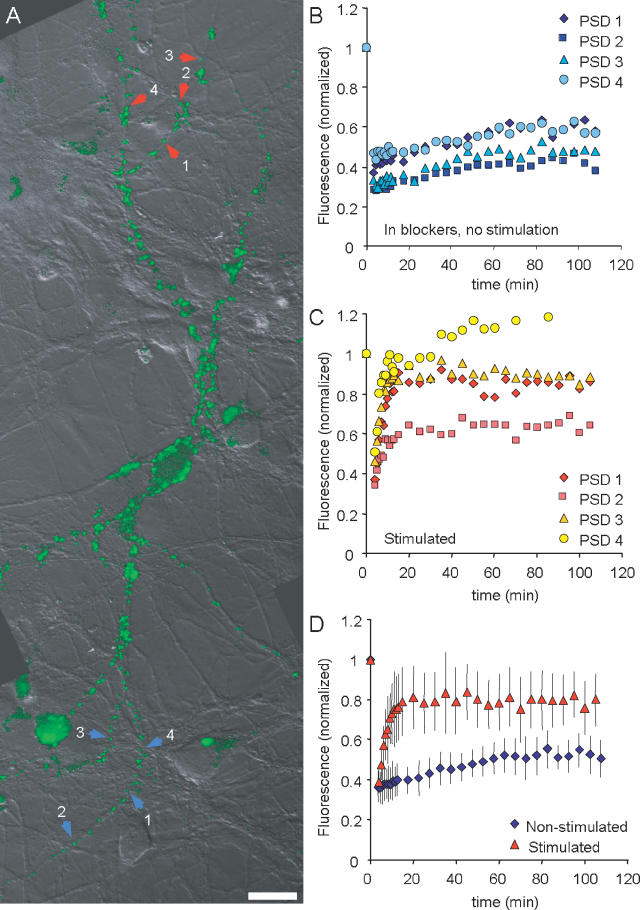
GFP:ProSAP2 Loss and Reincorporation Rates Are Accelerated by Synaptic Activity (A) A neuron (23 d in vitro) expressing GFP:ProSAP2 that was maintained for 7 d in glutamate receptor blockers (10 μM CNQX and 50 μM AP-5). Four PSDs (blue arrows at bottom of image) were photobleached and the recovery of fluorescence at these sites was monitored (B). Then, the preparation was switched to blocker-free solution and four other PSDs (red arrows at top of image) were photobleached. After collection of the first set of postphotobleach images, the preparations were stimulated at 20 Hz for 20 s every 3 min while monitoring the recovery of the photobleached PSDs (C). A comparison of mean recovery kinetics for both sets of photobleached PSDs (D) reveals that recovery kinetics were greatly accelerated by the stimulation protocol, indicating that the loss and reincorporation kinetics of ProSAP2 at individual PSDs are accelerated by synaptic activation. Note that PSD 4 from (C) was not included here as its final fluorescence exceeded its original level, indicating that the size of this PSD may have changed during this experiment. All data are shown after correcting for ongoing photobleaching as in Figure 3. Bar in (A), 20 μm.

Although rapid and reliable fluorescence recovery was observed in most such experiments (*n* > 10), in two experiments performed in rather mature neurons (more than 3 wk in culture) recovery seemed to occur much more slowly (data not shown). We suspected that this may have been related to low activity levels in these particular preparations (that typically exhibit significant spontaneous activity; data not shown), and thus we stimulated the neurons for 20 s at 20 Hz and repeated the photobleaching procedure on other GFP:ProSAP2 puncta belonging to the same neurons. Surprisingly, rapid fluorescence recovery was observed at these puncta, which suggested that ProSAP2 dynamics may be affected by the activity history of the particular neuron (and perhaps, synapse). We thus performed similar experiments in preparations in which spontaneous activity was suppressed by chronic exposure to the AMPA- and NMDA-type glutamate receptor antagonists CNQX and AP-5 for 7 d prior to the experiments (see Materials and Methods). These preparations were mounted on the microscope and perfused continuously with a physiological solution containing the aforementioned glutamate receptor antagonists. Four PSDs residing in one field of view were then photobleached and their recovery followed for about 2 h (Figure 4A and 4B). Then, the perfusion solution was switched to CNQX and AP-5 free physiological solution, and four PSDs residing at a different region of the same neuron were photobleached. Here, however, collection of the first postphotobleaching images was followed immediately by a stimulus train (20sec@20Hz) that was repeated every 3 min until the termination of the experiment (labeling of presynaptic sites with FM4–64). As shown in Figure 4B through 4D, the recovery kinetics of the two PSD populations differed radically: Whereas fluorescence recovery was very slow in the nonstimulated population, recovery in the stimulated population was rapid, resembling and sometimes exceeding the recovery rates observed in preparations in which spontaneous activity was not manipulated (as in Figure 3).

**Figure 5:**
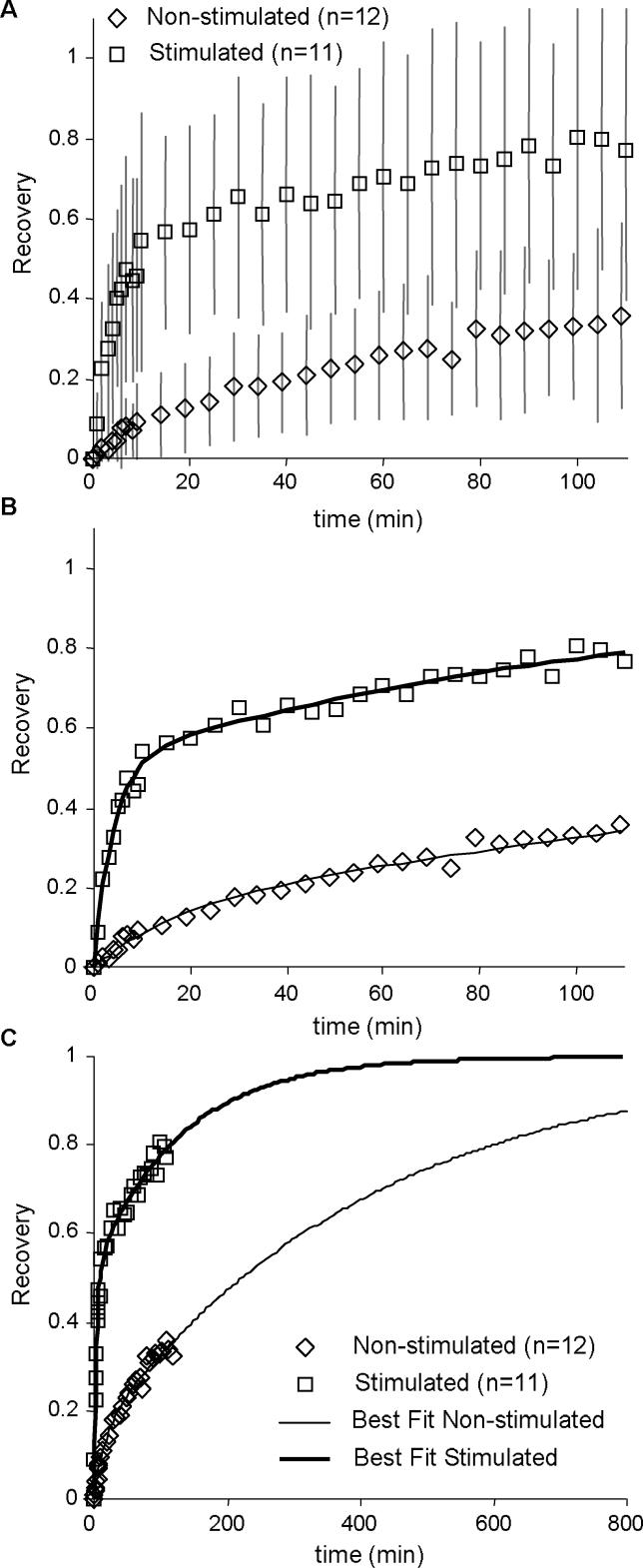
Synaptic Activity Accelerates GFP:ProSAP2 Loss and Reincorporation Rates (A) Mean recovery time course for all photobleached puncta in three separate experiments identical to that shown in Figure 4. Data shown are mean ± standard deviation for all photobleached puncta. (B) The data were fit according to a model that assumed two GFP:ProSAP2 pools with different recovery kinetics as described in the text. (C) Extrapolation of recovery time courses using the recovery rates and relative pool sizes that provided the best fit to the data.

The recovery kinetics for all photobleached PSDs in three identical experiments are summarized in Figure 5A. The pooled data strongly indicate that the recovery of GFP:ProSAP2 fluorescence is much faster following stimulation, with 63% recovery observed in the stimulated population after 43 min (*n* = 11) but only after about 350 min (extrapolated value) in the nonstimulated population (*n* = 12). However, as in the FRAP experiments described for Synapsin I above, considerable variability in the extent and kinetics of fluorescence recovery was observed, even among PSDs of the same neuron. To determine if activity had a statistically significant effect on the fluorescent recovery kinetics, we fit the recovery curve of each photobleached PSD to a recovery function that assumed two mobile pools with different recovery kinetics, and the aforementioned computer program was used to find the best fit to two time constants and the relative pool sizes. Differences between the time constants for the slow pool in the two groups did not reach statistical significance (434 ± 195 min nonstimulated, 409 ± 292 min stimulated). As for the fast pool, its small size in the nonstimulated population precluded a reliable comparison. On the other hand, the fractional size of the relatively mobile pool was significantly larger in the stimulated group (0.54 ± 0.27 stimulated versus 0.11 ± 0.12 nonstimulated, *p* < 0.0001, Student's one-tailed *t*-test). These findings indicate that the faster recovery kinetics observed following stimulation result from an increase in size of a relatively mobile pool of ProSAP2 at the expense of a less mobile pool.

### Redistribution of ProSAP2 among Nearby Dendritic Spines

Experiments presented above suggested that Synapsin I, once leaving a presynaptic site, may become incorporated into the presynaptic matrix of nearby boutons. Could PSD molecules display similar dynamics, or are they simply degraded as suggested previously [34,35]? To determine if this occurs, we substituted the GFP moiety of GFP:ProSAP2 with PA-GFP and transfected neurons with this fusion protein. Again, CFP was co-expressed to identify neurons expressing PA-GFP:ProSAP2. CFP fluorescence was then used to locate putative postsynaptic excitatory sites, identifiable as dendritic spines. Then, PA-GFP was activated along a short dendritic segment containing a group of dendritic spines by high-intensity illumination at 405 nm. Time-lapse imaging was then used to follow the redistribution of the photoactivated PA-GFP:ProSAP2 molecules. As we were interested to evaluate ProSAP2 redistribution in unperturbed conditions, no glutamate receptor blockers were present in these experiments.

**Figure 6:**
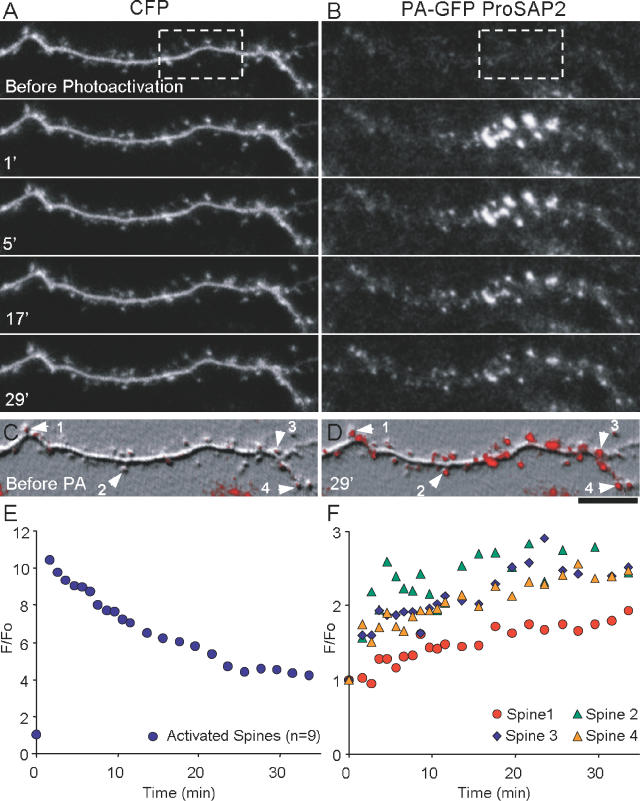
ProSAP2 Lost from Dendritic Spine Heads Is Incorporated into PSDs of Adjacent Spines A dendritic segment expressing both CFP (A) and PA-GFP:ProSAP2 (B). At time *t* = 0, PA-GFP:ProSAP2 within the region enclosed in rectangles was photoactivated by selective illumination at 405 nm. With time from photoactivation, fluorescence at tips of remote spines increased, whereas spine head fluorescence within the photoactivated region diminished. The contrast in (B) was enhanced linearly to emphasize fluorescence changes in remote spines, resulting in the apparent saturation at photoactivated spines. Spatial relationships between spines and PA-GFP:ProSAP2 puncta before and 29 min after photoactivation are shown in (C) and (D), respectively. In these images, PA-GFP:ProSAP2 fluorescence data were overlaid onto the CFP images after rendering the latter with the “emboss” filter of Adobe Photoshop. Note that PA-GFP:ProSAP2 fluorescence is restricted to spine heads, with little fluorescence observed in spine necks or the dendrite shaft. This distribution indicates that the PA-GFP:ProSAP2 that migrated to adjacent spines had become integrated into the PSD at these sites. Bar, 10 μm. Quantification of fluorescence changes at photoactivated (E) and neighboring spine heads (F) reveals a gradual decrease of fluorescence in the photoactivation region concomitant with fluorescence increases at nearby spines, most prominent at spines nearest to the photoactivation site. Values are normalized to prephotoactivation fluorescence levels.

One such experiment is shown in Figure 6. In this experiment, PA-GFP:ProSAP2 was activated along a approximately 15-μm dendritic segment that contained approximately ten spines and time-lapse fluorescence microscopy was then used to record the redistribution of the activated molecules. The fluorescence at the activated site decayed with kinetics similar to those observed for GFP:ProSAP2 in the FRAP experiments described above (63% decay within approximately 32 min; Figure 6E). Remarkably, however, as the fluorescence of the activated spines decayed, fluorescence increases were recorded in nearby spines at distances of up to 30 μm from the photoactivated segment (Figure 6B–6D and 6F), with the largest increases occurring near the activated segment, suggesting that ProSAP2 lost from the activated spines had migrated to nearby spines residing outside of the photoactivated segment. Interestingly, the increase in fluorescence was observed mainly in spine heads, not within the dendritic shaft, arguing against the possibility that the increased fluorescence resulted from a rise in the concentration of an inert, volume filling fluorescent substance, and supporting the possibility that the photoactivated PA-GFP:ProSAP2 had become incorporated into the PSDs of nearby spines.

The fluorescence increases observed in spines outside of the photoactivated region were relatively modest as compared to the large changes in fluorescence observed within the photoactivated segment. For example, in the experiment shown in Figure 6, photoactivation induced a 10-fold increase in PA-GFP:ProSAP2 fluorescence within spines in the photoactivated region, whereas fluorescence in spines outside this region increased by a factor of 2 to 3 after 30 min. Given that only about 60% of the photoactivated PA-GFP:ProSAP2 had dissociated from the photoactivated spines after 30 min, that nearby spines had exchanged about the same fraction of their own PA-GFP:ProSAP2 over the same time period, and that photoactivated PA-GFP:ProSAP2 may have been intermixing with nonphotoactivated PA-GFP:ProSAP2 from outside the activated segment, fluorescence increases of this magnitude were to be expected. Thus, these photoactivation experiments, together the FRAP experiments described above, strongly suggest that individual ProSAP2 molecules are continuously exchanged and redistributed among nearby PSDs and spines.

### Rate of Synapsin I and ProSAP2 Replenishment from Somatic Sources

The experiments described above indicate that molecules leaving synaptic structures can be reused and reassimilated into synaptic structures. However, synaptic proteins have finite lifetimes and thus must be eventually replaced if synapses are to be maintained, as failure to do so has severe consequences (see, for example, [36]). While some protein synthesis is carried out in dendrites and perhaps in axons too [37] the major site of protein synthesis is believed to be the neuronal cell body. What are the rates at which synaptic proteins from somatic source are supplied to and incorporated into remote synaptic structures? How do these replenishment rates compare with local dynamics, such as those described above?

To examine the rates at which synapses are replenished with proteins from somatic sources, we used PA-GFP–tagged variants of Synapsin I and ProSAP2 and measured the rates at which proteins photoactivated in the soma are trafficked to and incorporated into remote synaptic structures. To that end, neurons were transfected with CFP and PA-GFP:ProSAP2 or PA-GFP:Synapsin as described above. The axonal (for Synapsin) or dendritic (for ProSAP2) arbor of the neuron was mapped out using the robotic stage of our confocal microscope system, and an initial set of images was collected. Then, PA-GFP was activated in the soma by brief, high-intensity illumination at 405 nm. Time-lapse imaging was then used to follow the incorporation of the photoactivated molecules into synaptic structures.

**Figure 7:**
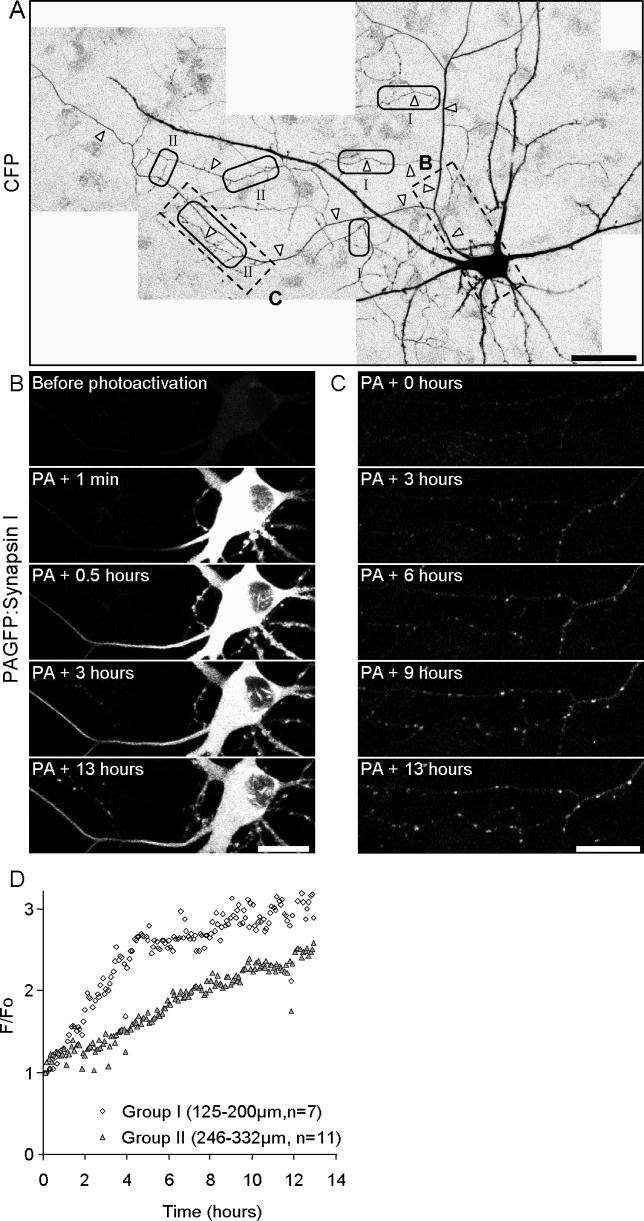
Incorporation Rates of Synapsin I from Somatic Sources at Remote Presynaptic Boutons (A) Low-magnification composite image of a neuron expressing CFP and PA-GFP:Synapsin I. Only CFP fluorescence is shown here. Parts of the axonal arbor are marked with arrowheads. Fluorescence is encoded using an inverted gray scale to increase detail clarity. (B) Higher magnification of rectangle marked as “B” in (A). At time *t* = 0, PA-GFP:Synapsin I within the cell body was photoactivated by selective illumination of the soma at 405 nm. Within minutes, photoactivated PA-GFP:Synapsin was observed to spread out into the axon and, much later, to appear at presynaptic boutons in the same field of view. (C) Higher magnification of rectangle marked as “C” in (A), enclosing a group of relatively remote presynaptic boutons. Note the gradual increase in fluorescence of these remote boutons over time. (D) Quantification of fluorescence changes at remote boutons following photoactivation of somatic PA-GFP:Synapsin. Boutons were grouped according to distance from the soma as shown in (A). Fluorescence data for each bouton were normalized to prephotoactivation fluorescence levels at the same bouton. Bars: (A), 50 μm; (B and C), 20 μm.

**Figure 8:**
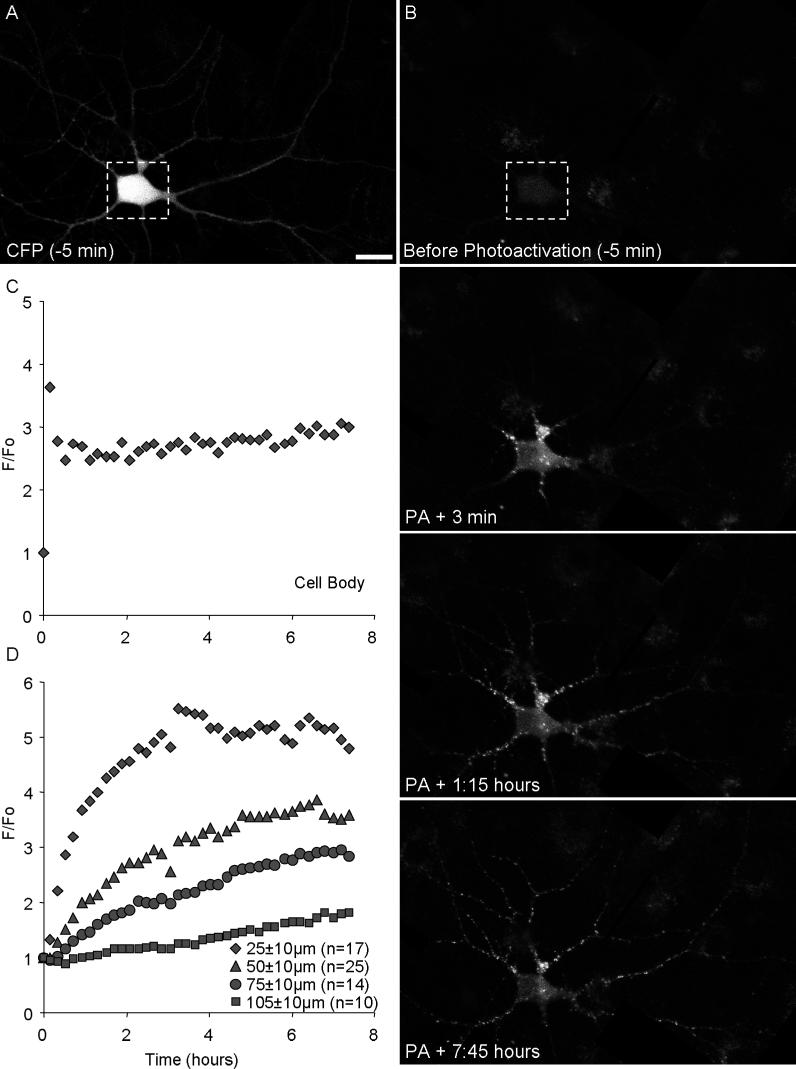
Incorporation Rates of ProSAP2 from Somatic Sources into Remote PSDs (A) Composite image of a neuron expressing CFP and PA-GFP:ProSAP2. Only CFP fluorescence is shown here. (B) PA-GFP:ProSAP2 fluorescence for same region as in (A). At time *t* = 0, PA-GFP:ProSAP2 within the cell body was photoactivated by selective illumination of the soma at 405 nm. With time, photoactivated PA-GFP:ProSAP2 migrated to PSDs along the dendrites, initially to proximal PSDs and later to more distal ones. During the time-lapse session, recurrent, low-level photoactivation of the soma was performed, to maintain a constant level of somatic, photoactivated PA-GFP:ProSAP2. (C) Quantification of fluorescence changes at the soma. (D) Quantification of fluorescence changes at PSDs, grouped according to distance from the soma. Fluorescence data for each PSD were normalized to prephotoactivation fluorescence levels for the same PSD. Bar, 20 μm.

Two examples of such experiments are shown in Figures 7 and 8. Figure 7A shows a low-magnification image of a neuron expressing PA-GFP:Synapsin I, as well as CFP, which was used to create this image. At time 0, PA-GFP:Synapsin I within the soma was photoactivated by 405-nm illumination confined to a region enclosing the soma (Figure 7B). As shown in Figure 7B and 7C, photoactivation was followed by a gradual and rather slow incorporation of the photoactivated protein into punctate structures along the axonal arbor. Quantifying the changes in fluorescence and the dependence on distance from the soma turned out to be somewhat difficult: First, axonal arbors were often quite complicated, with axonal branches crossing each other in such a fashion that it was difficult to reliably trace the route from some branches back to the soma. Secondly, over the long durations of these experiments, many Synapsin puncta merged with nearby puncta, split, or disappeared entirely, limiting the number of puncta for which valid measurements could be obtained (see also [28]). We thus grouped together data from relatively stable puncta located at similar distances from the soma. As shown in Figure 7D, the assimilation of Synapsin originating in the soma at stable puncta along the axonal arbor was a slow and protracted process and even after 14 h did not seem to be complete. Not surprisingly, assimilation rates were pronouncedly slower at regions further away from the soma.

Similar findings were observed in 6 separate experiments, that is, slow accumulation of photoactivated PA-GFP:Synapsin I at puncta along axons, with the slowest and most protracted accumulation recorded at the most remote puncta. Due to effects of axonal geometry on trafficking kinetics (both diameter and branching patterns) we did not attempt to pool the data from these experiments. However, accumulation kinetics of photoactivated material at remote puncta sites was qualitatively similar, reaching a plateau at distances of 300 to 500 μm only after 18 to 24 h. FM4–64 labeling performed at the end of three experiments confirmed that the majority of stable puncta in which photoactivated PA-GFP:Synapsin I was observed to accumulate represented functional presynaptic sites (Figure S2A).

Similar experiments were performed with PA-GFP:ProSAP2 (Figure 8). Here, however, we noted that a single photoactivation episode was not sufficient to maintain a high and constant level of photoactivated material within the soma. Thus, during these experiments, the soma was briefly reactivated every 6 to 10 min in order to maintain a constant level of photoactivated material within this region (Figure 8C). Here, too, we observed a slow accumulation of photoactivated PA-GFP:ProSAP2 at punctate structures (presumably PSDs) along dendritic arbors, with the slowest and most protracted accumulation recorded at the most remote PSDs (Figure 8B and 8D). For example, in the experiment shown in Figure 8, assimilation of photoactivated PA-GFP:ProSAP2 into PSDs residing at distances of approximately 25 μm plateaued after approximately 3 to 4 h, but at distances of approximately 100 μm from the soma the assimilation process was much slower, and was not nearly complete even after 8 h. Qualitatively similar results were observed in all such experiments (*n* = 6). FM4–64 labeling performed at the end of 2 experiments revealed that most punctate sites of photoactivated PA-GFP:ProSAP2 accumulation were juxtaposed against functional presynaptic boutons (Figure S2B), indicating that these puncta represented bone fide postsynaptic structures.

In these experiments, fluorescence levels of photoactivated PA-GFP–tagged Synapsin or ProSAP2 in the soma were maintained at approximately constant levels (see, for example, Figure 8C). Under these conditions, steady state fluorescence levels at remote (approximately 100 μm) PSDs would be expected to ultimately reach steady state levels similar to those recorded at proximal (approximately 25 μm) PSDs. If this assumption is correct, the remote PSDs would have exchanged only about 10% of their PA-GFP:ProSAP2 content with material arriving from the soma over a period of 4 h (Figure 8D). On the other hand, the FRAP experiments described above indicate that nearly the entire GFP:ProSAP2 content of individual PSDs is exchanged over the same time period (Figure 5C). Similarly, only about 5% of the PA-GFP:Synapsin I content of remote (approximately 300 μm) boutons would have been replaced with material arriving from the soma (Figure 7D) over the same time period during which nearly the entire GFP:Synapsin I content of presynaptic boutons is exchanged (about 1 h; Figure 1E and 1F). This mismatch between synaptic exchange rates and somatic replenishment rates indicates that most of the protein reincorporated into photobleached synapses comes from local sources, that is extrasynaptic material and, in all likelihood, neighboring synapses. Accordingly, the experiments described here (Figures 7 and 8) provide estimates of the rates at which these local pools are replenished with material arriving from somatic sources.

### Effects of Protein Synthesis and Degradation Inhibitors on Synapsin I and ProSAP2 Exchange Rates

The photoactivation experiments described above (Figures 2 and 6) indicate that Synapsin I and ProSAP2 lost from one synaptic site can be assimilated into neighboring synaptic structures. We noted, however, that the total fluorescence of axonal or dendritic segments within which synaptic structures were photoactivated was not conserved and it gradually diminished over time (data not shown). This could represent the migration of photoactivated material out of the field of view, but it could also indicate that some of the material lost from synaptic structures was rapidly degraded. Along the same lines, material incorporated into photobleached synaptic structures could have come from preexisting protein pools (extrasynaptic and synaptic alike) but it could also consist of new material synthesized locally or delivered directly from somatic biosynthetic centers (at least for proximal synapses).

**Figure 9:**
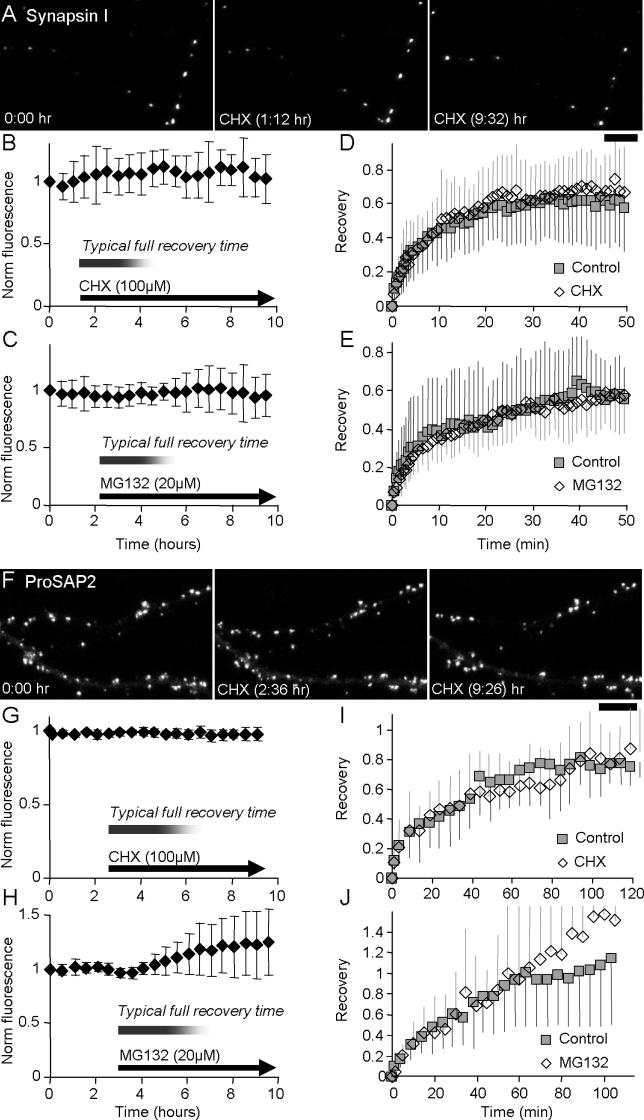
Effects of Cycloheximide and MG132 on Steady State Levels and Exchange Rates of GFP:Synapsin I and GFP:ProSAP2 (A) Long-term imaging of axons of neurons expressing GFP:Synapsin I, before and after the addition of cycloheximide (CHX). CHX was added to attain a final concentration of 100 μM 1 h 12 min after the beginning of the experiment. (B) Mean fluorescence levels of all GFP:Synapsin puncta recorded in this experiment normalized to their mean fluorescence intensities at the beginning of the experiment (five cells, 13 fields of view, 197 synaptic boutons). (C) A similar experiment in which MG132 was added 2 h 29 min after the beginning of the experiment to attain a final concentration of 20 μM. Fluorescence levels were normalized to fluorescence intensities at the beginning of the experiment (four cells, ten fields of view, 225 synaptic boutons). (D) Paired FRAP experiments were performed in the same preparations of neurons expressing GFP:Synapsin I, first in the absence and then in the presence of cycloheximide (three experiments, 12 boutons before cycloheximide addition, 11 boutons after cycloheximide addition). (E) Paired FRAP experiments as in (D) but with MG132 instead of cycloheximide (four experiments, 13 boutons before MG132 addition, 16 boutons after MG132 addition). All experiments in (A–E) were performed in CNQX and AP-5 to avoid the confounding effects of spontaneous activity. (F) Long-term imaging of dendrites of neurons expressing GFP:ProSAP2, before and after the addition of cycloheximide (at 2 h 36 min after the beginning of the experiment). (G) Mean fluorescence levels of all GFP:ProSAP2 puncta recorded in this experiment normalized to their mean fluorescence intensities at the beginning of the experiment (five cells, five fields of view, 678 PSDs). (H) A similar experiment in which MG132 was added 3 h 4 min after the beginning of the experiment (seven cells, eight fields of view, 574 PSDs). (I) Paired FRAP experiments were performed in neurons expressing GFP:ProSAP2, first in the absence and then in the presence of cycloheximide (three experiments, ten PSDs before cycloheximide addition, eight PSDs after cycloheximide addition). (J) Paired FRAP experiments as in (I) but with MG132 instead of cycloheximide (three experiments, nine PSDs before MG132 addition, nine PSDs after MG132 addition). Data in FRAP experiments were not corrected for photobleaching during the recovery phase to avoid canceling out general changes in puncta fluorescence related to cycloheximide or MG132 addition. For sake of clarity, only one-sided error bars are shown in (D, E, I, J). Bars (A, F), 10 μm.

To examine the contribution of protein synthesis and degradation to Synapsin I and ProSAP2 exchange rates over the time-scales of the experiments described above, we performed two types of experiments. In the first set we examined if steady state levels of GFP-tagged Synapsin I and ProSAP2 at synapses are affected by pharmacological agents that inhibit protein synthesis or proteasome-mediated protein degradation, with the expectation that these manipulations should reduce or increase the GFP:Synapsin I and GFP:ProSAP2 content of synapses if protein synthesis/degradation contribute significantly to exchange dynamics over the time-scales determined in FRAP experiments (1 to 4 h). To that end, neurons expressing GFP:Synapsin or GFP:ProSAP2 were imaged continuously at 30 min intervals. After collecting baseline images for 1.5 to 3 h, the protein synthesis inhibitor cycloheximide (100 μM) or the proteasome-mediated degradation inhibitor MG132 (20 μM) was added to the media, and images were collected for another 6 h or longer (Figure 9). As shown in Figure 9A through 9C, these treatments had virtually no effect on the steady state levels of GFP:Synapsin I during this period (nine cells, two separate experiments). Steady state levels of GFP:ProSAP were not affected by cycloheximide either (Figure 9F and 9G; ten cells, two separate experiments). The effects of MG132 on GFP:ProSAP steady state levels were mixed: In about half of neurons examined here (seven of 12), GFP:ProSAP levels were not affected at all by the addition of MG132. However, in others (five of 12) we did observe a gradual increase in GFP:ProSAP puncta intensity (approximately 50% over 6 to 10 h; see also [34,35]).

In the second set of experiments we performed two consecutive FRAP experiments (as in Figure 4) in neurons expressing GFP:Synapsin or GFP:ProSAP2, first in the absence and then after the addition of either cycloheximide or MG132. As shown in Figure 9D, 9E, 9I, and 9J, fluorescence recovery kinetics were not affected by the addition of cycloheximide or MG132 in any of these paired experiments. However, the fluorescence of some GFP:ProSAP2 puncta did increase gradually beyond their initial fluorescence levels (Figure 9J), in line with an overall increase in GFP:ProSAP puncta intensity associated with proteasome-mediated degradation blockade (by about 10% to 15% over the time-scale of full recovery in FRAP experiments; Figure 9G). These findings thus indicate that the protein dynamics recorded in the FRAP and photoactivation experiments of Figures 1 to 6 mainly reflect processes of exchange and redistribution rather than bona fide protein turnover, i.e., protein synthesis and degradation, although these findings also suggest that local ProSAP2 pool sizes may be subject to regulation by proteasome-mediated degradation (see also [34,35]).

## Discussion

As a step toward a better understanding of processes involved in synaptic maintenance, we have examined the loss, redistribution, reincorporation, and replenishment dynamics of Synapsin I and ProSAP2/Shank3, two prominent components of the presynaptic matrix and PSD, respectively. The experiments described above strongly indicate that both these molecules are continuously lost from, redistributed among, and reincorporated into synaptic structures at time-scales of minutes to hours. These exchange rates were not affected by acute inhibition of either protein synthesis or proteasome-mediated protein degradation, could be accelerated by stimulation, and greatly exceeded the rates of replenishment from somatic sources, particularly at remote synapses. These findings indicate that the dynamics of key synaptic matrix proteins, at least insofar as Synapsin I and ProSAP2 are concerned, are dominated by local protein exchange and redistribution whereas protein synthesis and degradation seem to be second-order processes that serve to maintain and regulate the size of the local synaptic protein pools.

### Exchange and Redistribution as Dominant Determinants of Synaptic Matrix Protein Dynamics

The FRAP experiments described above (Figures 1, 4, 5, and 9) reveal that Synapsin I and ProSAP2 are continuously lost from and reincorporated into individual synaptic structures, a conclusion supported by the decay kinetics of photoactivated PA-GFP–tagged variants of the same molecules (Figures 2D and 6E). Interestingly, both molecules exhibited biphasic fluorescence recovery kinetics, which points to the existence of at least two protein pools with different exchange rates (see also[20]). At the moment, we do not know what each pool represents. However, one very unlikely possibility is that fast pools represent freely diffusible cytosolic molecules. As fast as these exchange rates may seem (τ = 2 to 5 min), they are much slower than those of freely diffusible molecules (see also Figures S1B and 9D). Indeed, in control experiments in which FRAP was performed in neurons expressing EGFP alone (not fused to any protein), recovery was essentially instantaneous at the fastest sampling rates used here (12-s intervals; data not shown), in good agreement with other studies [20,38]. Similarly, in cells expressing PA-GFP alone, photoactivated PA-GFP dispersed almost immediately when photoactivated at individual synaptic locations (data not shown). Thus, both “fast” and slow pools represent molecules in bound states, although the differences between these states are not known.

Photoactivation of PA-GFP:Synapsin I and PA-GFP:ProSAP2 clustered at synaptic sites revealed that molecules lost from photoactivated synapses may migrate away from these synapses and subsequently become reincorporated into nearby synaptic structures. These findings imply that Synapsin I and ProSAP2 are constantly exchanged among nearby synapses, in essence forming local protein pools shared by neighboring synaptic structures. Are these dynamics unique to Synapsin and ProSAP2, or are they characteristic of synaptic cytomatrix proteins in general? As only two proteins were studied here, this is yet to be determined. It is worth pointing out that the ability to detect the redistribution of photoactivated molecules depends on several factors, including their loss and reincorporation rates, their diffusion/transport rates, the extrasynaptic volume, and additional geometric factors. For example, if loss and reincorporation rates are low, effective diffusion rates high and the extrasynaptic volume large, the fraction of photoactivated material incorporated into nearby synapses could be too low to be detected. In this respect, the selection of Synapsin I and ProSAP2 was somewhat fortuitous, as these molecules exhibited relatively high loss and reincorporation rates, and slow effective diffusion rates (probably due to interactions with cytoskeletal elements within axons and dendrites). Yet, previously published data hints that the redistribution dynamics displayed by Synapsin I and ProSAP2 are not unique: For example, FRAP experiments suggest that many postsynaptic molecules (PSD-95, SAP97, PSD-Zip45/Homer1c, alpha-Actinin; Neurabinl, Actin, brain-enriched GK domain-associated protein, ProSAP1, AMPA receptor subunit 1, NMDA receptor subunit 1, CaMKII, N-Cadherin) are lost from and reincorporated into individual synapses at rates that are similar or much higher than those reported here for Synapsin I or ProSAP2 [14–20,38–40]. At the presynaptic side, FRAP experiments have shown that synaptic vesicle membrane proteins (VAMP, synaptotagmin) are exchanged among individual synapses [27] whereas a recent study has demonstrated that even entire synaptic vesicles are exchanged among and shared by nearby presynaptic boutons [28]. Although yet to be shown experimentally, it seems likely to us that other, perhaps the majority of synaptic cytomatrix proteins that exhibit significant lost and incorporation rates in FRAP experiments also exhibit redistribution dynamics similar to those reported here for Synapsin I and ProSAP2.

### Slow Replenishment of Local Synaptic Protein Pools from Somatic Sources

In comparison with the fairly rapid loss and incorporation rates of GFP-tagged Synapsin I and ProSAP2 at individual synapses, the rates at which synapses incorporated somatically photoactivated PA-GFP:Synapsin I or PA-GFP:ProSAP2 were much slower (Figures 7 and 8). The latter incorporation kinetics provide estimates of the rates at which local Synapsin I or ProSAP2 pools are replenished with material arriving directly from somatic sources. If PA-GFP:Synapsin and PA-GFP:ProSAP2 are synthesized primarily in the cell body, however, these estimates may be exaggerated to some degree due to the fact that the photoactivation procedure we used resulted in the activation of *all* PA-GFP–tagged proteins within the soma, not only of recently synthesized proteins. On the other hand, if somatic protein synthesis and export rates greatly exceed the repetition rates of the photoactivation procedure, a possibility exists that a pool of somatically derived material could have escaped photoactivation, which could lead, in principal, to underestimates of replenishment rates. However, the indifference of steady state GFP:Synapsin I and GFP:ProSAP2 levels to protein synthesis inhibition over many hours (Figure 9A, 9B, 9F, and 9G) strongly argues against this possibility. Thus the relatively slow incorporation kinetics of somatically photoactivated material (Figures 7 and 8) as compared to the exchange kinetics recorded in FRAP experiments further support the conclusion that the molecular dynamics of synaptic matrix proteins are dominated by local processes.

The incorporation of PA-GFP–tagged Synapsin I was noticeably slower and protracted the further away synapses were from the soma. This gradient indicates that synaptic proteins trafficked to distal axonal regions are continuously incorporated into and lost from synapses *en route,* resulting in a preferential replenishment of proximal synapses. Given that axons *in vivo* can reach extraordinary lengths (much greater than those observed in culture) and that it took more than half a day to replace most of the PA-GFP:Synapsin at “remote” synapses (approximately 300 to 500 μm from the soma), the replenishment of synapses many centimeters away from the soma would be expected to take many days, perhaps weeks. Of course, many presynaptic proteins are trafficked at much faster rates on vesicular carriers. Indeed, Synapsins can be carried on vesicular organelles [41,42]. Yet, studies carried out in retinal ganglion cells indicate that this fraction is relatively insignificant, as >90% of Synapsin is transported as part of the slow component B of axonal transport [41,43] and that the transport of Synapsin from the retina to the superior colliculi (approximately 1.2 cm in mice, approximately 3.5 cm in rabbits) takes about 7 to 8 d, in general agreement with our findings.

In comparison to axons, dendrites are typically much shorter, and thus would seem to pose less severe trafficking challenges. Yet dendrites can reach considerable lengths (many hundreds of microns). As our experiments indicate that the incorporation of PA-GFP:ProSAP2 from somatic sources into “remote” PSDs (approximately 100 μm from the soma) was not nearly complete even after 8 h (Figure 8), the complete replenishment of truly remote postsynaptic sites with ProSAP2, and perhaps other molecules synthesized in the soma would be expected to take days. This conclusion does not concur with the rather short life spans reported for many postsynaptic molecules (time constants ≪ 40 h [34]), and observations that ProSAP2 is degraded at significant rates (Figure 9; see also [34,35]). Perhaps these constraints impose a requirement for a distributed somatodendritic protein synthesis system [37,44,45]. Indeed, mRNAs of Shanks/ProSAPs [46] as well as those of additional PSD molecules [37] have been located in dendrites. On the other hand, it remains possible that ProSAP2 trafficking is atypically slow or that PSD molecules are protected somehow while in transit [35]. Thus, important questions as to how neurons manage to maintain their most remote PSDs remain open, and further experiments are necessary to resolve these issues (see also [47]).

### Synaptic Activity Can Accelerate Loss and Reincorporation of Synapsin I and ProSAP2

Brief episodes of synaptic activity were observed to accelerate the loss and reincorporation rates of both GFP:Synapsin I and GFP:ProSAP2 (Figures 1, 4, and 5) mainly by increasing the relatively mobile pools sizes of these proteins. Given that presynaptic activity is associated with Synapsin I dispersion [9,10] (Figure 2), the effects of activity on the Synapsin I dynamics were not surprising. However, we did not observe a direct effect of stimulation on GFP:ProSAP2 distribution (such as dispersion) and thus the acceleration of GFP:ProSAP2 loss and reincorporation kinetics by activity was unexpected.

Do these findings imply that synaptic activity can act in some cases as a “dispersive” force that promotes weakening of molecular interactions that bind synaptic molecules together? This notion is supported by quite a few studies. For example, clusters of acetylcholine (ACh) receptors at neuromuscular junctions are dispersed by presynaptic secretion of ACh [48,49]. Similarly, glutamate or glutamate receptor agonists can increase the mobility of proteins within dendritic spine membranes [50], increase the lateral mobility of AMPA receptors at glutamatergic synapses, accelerate their removal from postsynaptic membranes [51–53] and disrupt their associations with transmembrane AMPA receptor regulatory proteins [54]. Conversely, prolonged (days) blockade of activity or synaptic transmission reduces the mobility of AMPA receptors [52] and increases the concentration of both AMPA receptors [55] and NMDA receptors [56] at postsynaptic membranes. Stimulation was also shown to induce the dispersal of postsynaptic scaffolding proteins such as PSD-Zip45/Homer 1c [14] and SAP97 [18]. Interestingly, stimulation patterns similar to those used here were previously shown to reduce presynaptic function, perhaps by promoting the loss of synaptic molecules from presynaptic sites [57].

### Concluding Remarks

In considering the experiments reported here, several issues warrant discussion. The first is the fact that both proteins examined here were tagged with GFP, implying that an approximately 30-kDa polypeptide was added to the N-termini of these proteins. Although the use of GFP fusion proteins is commonplace nowadays, the possibility remains that this tag interferes, perhaps in subtle ways, with the interactions of Synapsin I or ProSAP2 with their endogenous binding partners and consequently alters and perhaps exaggerates their apparent dynamics. Furthermore, as the expression of both proteins is based on transfected cDNA the importance of dendritic (and axonal?) protein synthesis may have been underestimated due to the potential lack of appropriate mRNA targeting signals (this is particularly relevant for GFP:ProSAP2). Unfortunately, the use of GFP fusion proteins is still the best methodology available to date for studying protein dynamics in living cells, and while alternative methods are being developed, they are not without their own sets of problems [58].

A second issue relates to the preparation used here. Aside from the fact that the experiments were performed in a reduced system (cell culture), they were performed in neurons that are relatively immature (2 to 4 wk in vitro) compared to those in the mature rat brain. Given that recent in vivo imaging studies indicate that maturation is associated with decreases in dendritic spine dynamics [2,3] it is possible that other types of synaptic dynamics, such as those addressed here, are also subdued with maturation.

However, if our findings turn out to be correct and if the dynamics exhibited by Synapsin I and ProSAP2 turn out to be characteristic of synaptic cytomatrix proteins in general, they raise intriguing questions as to how synapses manage to maintain their structural and functional identity in face of these dynamics. For example, it has often been assumed that activity-induced changes to synaptic function are synapse-specific, that is, independent of changes at other synaptic connections. If activity-induced potentiation of synaptic function involves posttranslational modifications of postsynaptic molecules and if modified proteins subsequently redistribute among neighboring postsynaptic sites, it is hard to imagine how synapse specificity could be retained over long durations. Indeed, synaptic specificity is not always conserved [59]. For example, the synapse specificity of long-term potentiation was reported to break down at distances of about 70 μm [60]. Furthermore if, as suggested above, some forms of activity weaken molecular interactions within synaptic matrices, while others promote opposite effects [20,48,61–66], it is not hard to imagine how nearby synapses that share a limited pool of synaptic proteins could compete for these proteins and consequently, for synaptic strength [67–69]. Finally, and perhaps most fundamentally, if synaptic cytomatrices are so inherently dynamic, how do synapses retain their overall organization and function for weeks, months, and perhaps longer [1–3]? Phrased differently, if synaptic molecules are in a dynamic equilibrium with extrasynaptic pools, what drives and maintains their high concentrations at synaptic locations? Although one could envision a scenario in which extremely stable synaptic cytomatrix components serve as “nuclei” that define the location and perhaps the characteristics of synaptic scaffolds, even relatively stable synaptic molecules such as PSD-associated CaMKII [48] are exchanged over time-scales of minutes to hours [20]. At present, answers to these questions and even the questions themselves are somewhat speculative. Yet given the remarkable molecular dynamics exhibited by synaptic cytomatrix molecules studied so far, it may be fair to conclude that the ability of synapses to maintain some level of structural and functional stability over long durations is no less wondrous than their capacity for activity induced change and plasticity.

## Materials and Methods

### Cell culture.

Hippocampal cell cultures were prepared from 1- to 2-d-old Wistar rats as described previously [17] except that neurons were plated in media containing 10% NuSerum (Becton Dickinson Labware, Bedford, Massachusetts, United States) instead of 10% fetal calf serum. Cultures were maintained at 37 °C in 95% air/5% CO_2_ humidified incubator, and culture media was replaced every 7 d. For some experiments (Figures 4 and 5), neurons were maintained for 7 d before use in CNQX and AP-5 (added every 2 d from 2,000× and 1,000× stock solutions in DMSO and distilled water, respectively) reaching final concentrations of 10 to 30 and 50 to 150 μM, respectively. Cycloheximide (Sigma, St. Louis, Missouri, United States) and MG-132 (Calbiochem, San Diego, California, United States) were added from 3,400× and 500× stock solutions in DMSO to reach final concentrations of 100 μM and 20 μM, respectively.

### DNA constructs and expression.

GFP:Synapsin I [9] was provided as a generous gift by Dr. Timothy A. Ryan (Weill Medical College of Cornell University, New York, New York, United States). GFP:ProSAP2 (Shank3) was prepared as described previously [17]. PA-GFP:Synapsin was prepared by cutting Synapsin I out of GFP:Synapsin I with BglII and SacI and inserting it into PA-GFP-C1, provided as a generous gift by Dr. Lippincott-Schwartz (National Institute of Child Health and Human Development, Bethesda, Maryland, United States). PA-GFP:ProSAP2 was prepared by subcloning the ORF of the ProSAP2/Shank3 [17] into a specially prepared PA-GFP-C2 vector. Expression of GFP-tagged proteins was performed by calcium phosphate transfection as described previously [17] on days 7 to 10 in vitro. Coexpression of PA-GFP–tagged proteins and CFP was performed in a similar fashion using a DNA mixture prepared by mixing the DNA for these molecules at ratios of 5:1 (PA-GFP:Synapsin I:CFP) and 8:1 (PA-GFP:ProSAP2:CFP), followed by ethanol precipitation and resuspension in distilled water.

### Microscopy.

Scanning fluorescence and DIC images were acquired using a custom designed confocal laser scanning microscope based on a Zeiss Aviovert 200 using a ×40 1.3 N.A. Fluar objective. The system is controlled by software written by one of us (NEZ) and includes provisions for automated, multisite time-lapse microscopy. CFP and EGFP/FM4–64 were excited using the 457- and 488-nm lines of an argon laser respectively. Fluorescence emissions were read using 487- to 517- and 500- to 545-nm band-pass and >630-nm long-pass filters, respectively (Chroma, Vermont, United States). Time-lapse recordings were usually carried out by averaging three to six frames collected at each of two to seven focal planes spaced 0.7 to 0.9 μm apart. All data were collected at 640 × 480 resolution, at 12 bits per pixel, with the confocal aperture fully open. For long-term experiments (Figures 7 and 8), preparations were imaged within their cloning cylinders in the presence of their growth media (no perfusion). The coverslips were mounted in a modified heated chamber (Warner Instrument Corp.), placed in a custom-designed enclosure flooded with a sterile mixture of 5% CO_2_ and 95% air. The chamber and objective were heated to 37 to 38 °C using resistors and thermal foil and were controlled separately. This setup resulted in stable intrachamber temperatures of 33 to 35 °C. Data were collected sequentially from multiple predefined sites along the axonal and dendritic arbors, using the microscope systems robotic XYZ stage to cycle automatically through these sites at predetermined time intervals. Focal drift was corrected automatically using the microscope systems “autofocus” feature. For experiments requiring media changes and stimulation, the cloning cylinder was removed and cells were perfused with preheated Tyrode's saline solution (119 mM NaCl, 2.5 mM KCl, 2 mM CaCl_2_, 2 mM MgCl_2_, 25 mM HEPES, 30 mM glucose, buffered to pH 7.4) with or without CNQX (10 μM) and AP-5 (50 μM) as described in text. Stimulation was performed by passing 1-ms, 18-mA current pulses through platinum electrodes placed on both sides of the chamber. FM4–64 labeling was performed by flooding the perfusion chamber with Tyrode's containing 15 μM FM4–64 (Invitrogen, Carlsbad, California, United States) stimulating the neurons to fire action potentials (30 s at 10 Hz), leaving the dye in for an additional 30 s and rinsing with Tyrode's saline for 8 to 10 min. Dye unloading was performed by stimulating the neurons for 120 s at 10 Hz.

Photobleaching was performed by defining three to five 12 × 12 pixel (approximately 1.5 × 1.5 μm) regions of interest in each field of view and scanning them sequentially and repeatedly at 488 nm at high illumination intensity using the confocal microscope systems AOTF. This procedure was performed programmatically using Visual Basic for Applications from within Microsoft Excel to control the microscope system via OLE automation. Photoactivation was performed by selectively scanning rectangular regions of interest surrounding the photoactivated objects (boutons, spines, cell bodies) at 405 nm using a violet diode laser (Coherent).

### Image analysis.

All data analysis was performed using software (“OpenView”) written for this purpose by one of us (NEZ). Analysis was performed on maximal intensity projections of Z section stacks. Intensities of fluorescent puncta were measured by centering 8 × 8 to 12 × 12 pixel boxes on them, and obtaining the mean pixel value in this rectangular region. FRAP data were normalized and corrected for ongoing photobleaching according to the following equation

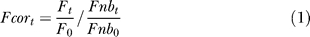
where *F_t_* is the fluorescence at time *t*, *F_0_* is the prephotobleaching fluorescence, *Fnb_t_* is the average fluorescence intensity of five to ten nonbleached puncta at time *t,* and *Fnb_0_* is the average fluorescence intensity of the same nonbleached puncta at time *t* = 0. Photoactivation data were normalized to fluorescence intensities measured at the same structures before photoactivation.

Best fits of FRAP recovery curves were made according to the following equation

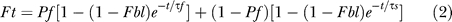
where *P_f_* is the relative size of the fast pool (expressed as a fraction of 1), *F_bl_* is the normalized fluorescence immediately after the photobleaching procedure, and *τ_f_* and *τ_s_* are the recovery time constants for the fast and slow pools, respectively. A program was written in Visual Basic for Applications within Microsoft Excel that explored systematically a wide range of *P_f_* , *τ_f_*, and *τ_s_* combinations and obtained the values that gave the best fit to the experimental data (minimal sum of squared residuals).

Images for figures were processed by linear contrast enhancement and low-pass filtering using Adobe Photoshop and prepared for presentation using Microsoft PowerPoint.

## Supporting Information

Figure S1 High–Temporal Resolution FRAP Experiments Line scan imaging was used to resolve fluorescence recovery during the first few seconds following a bleach procedure. To that end, a single line passing through a structure of interest was scanned consecutively 480 times (approximately 4.6 ms/line). At line 20 the laser illumination intensity was stepped abruptly from 2% to 100%, 20 lines were scanned and then illumination intensity was restored to 2%. Fluorescence values represent the average fluorescence of pixels along the line segment residing within the structure of interest.
(A and C) GFP:Synapsin I in individual boutons (A, average for 15 boutons) and GFP:ProSAP2 at individual PSDs (C, average of 18 PSDs).
(B and D) GFP:Synapsin I in axonal segments (B, average of seven separate segments) and GFP:ProSAP2 in dendritic segments (D, average of four separate segments). Note that within the time frame of these experiments, the fluorescence seemed to recover to levels that were lower than prephotobleach fluorescence levels. This may indicate that even within axons and dendrites, diffusion of some GFP:Synapsin I and GFP:ProSAP2 molecules is retarded by interactions with relatively immobile elements such as actin microfilaments. However, it should also be noted that as baseline fluorescence levels in dendritic and axonal segments were relatively low, higher laser illumination intensities (5%) were required to obtain acceptable signals, and that these illumination intensities were associated with significant ongoing bleaching. Using rates of ongoing bleaching obtained in control experiments and a discrete element model for FRAP in neurites that also accounts for ongoing bleaching (NEZ, unpublished data), we found that the experimental data can be explained well by diffusion and ongoing bleaching using diffusion constants of 1.1 × 10^−8^ cm^2^/s and 0.9 × 10^−8^ cm^2^/s for GFP:Synapsin and GFP:ProSAP2, respectively (red lines).
(68 KB EPS)

Figure S2 Somatically Photoactivated PA-GFP–Tagged Molecules Accumulate at Synaptic Sites (A) An axonal segment of a neuron expressing CFP and PA-GFP:Synapsin I, located approximately 100 μm from the cell body. At time *t* = 0, PA-GFP:Synapsin I in the soma was photoactivated (as in Figure 7) and the segment was followed by automated time-lapse confocal microscopy. Bar, 5 μm.
(B) Gradual accumulation of photoactivated PA-GFP:Synapsin at a varicosity along the axon.
(C) Labeling with FM4–64 at the end of the experiment revealed that sites at which photoactivated PA-GFP:Synapsin had accumulated (arrowheads) exhibited a capacity for evoked FM4–64 uptake and release, indicating that these represented functional presynaptic boutons.
(D) A dendritic segment of a neuron expressing CFP and PA-GFP:ProSAP2, located approximately 45 μm from the cell body. At time *t* = 0, PA-GFP:ProSAP2 in the soma was photoactivated (as in Figure 8) and the segment was followed by automated time-lapse confocal microscopy. Bar, 5 μm.
(E) Gradual accumulation of photoactivated PA-GFP:ProSAP2 at punctate structures along the dendrite.
(F) Labeling with FM4–64 at the end of the experiment revealed that most puncta at which photoactivated PA-GFP:ProSAP2 had accumulated (arrowheads) were juxtaposed against functional presynaptic sites, indicating that these puncta constituted postsynaptic sites.
(831 KB EPS)

## Abbreviations

AOTF: acousto-optical tunable filter
AP-5: 2-amino-5-phosphonopentanoic acid
AZ: active zone
CAZ: cytoskeletal matrix associated with the active zone
CFP: cyan fluorescent protein
CNS: central nervous system
CNQX: 6-cyano-7-nitroquinoxaline-2,3-dione
EM: electron microscope
FRAP: fluorescence recovery after photobleaching
GFP: green fluorescent protein
PA: photoactivatable
PSD: postsynaptic density
